# Prescribed therapy for asthma: therapeutic ratios and outcomes

**DOI:** 10.1186/s12875-015-0265-2

**Published:** 2015-04-14

**Authors:** Laurent Laforest, Idlir Licaj, Gilles Devouassoux, Irene Eriksson, Pascal Caillet, Gérard Chatte, Manon Belhassen, Eric Ganse

**Affiliations:** Pharmacoepidemiology Lyon, UMR 5558 CNRS - Claude Bernard University, Lyon, France; Respiratory Medicine, Croix Rousse University Hospital, Lyon, France; Centre for Pharmacoepidemiology, Department of Medicine, Karolinska Institutet, Stockholm, Sweden; Epidemiology and Public Health Department, Amiens University Hospital Center, Amiens, France; Respiratory physician, Caluire, France

**Keywords:** Electronic health records, Primary care, Asthma, Inhaled corticosteroids, Prescribing, Exacerbations

## Abstract

**Background:**

Inhaled corticosteroids (ICS) are the cornerstone of asthma therapy. The ICS-to-total-asthma-medication ratios, calculated from claims data, indicate potentially risky disease management in asthma. Our aim was to assess the utility of ICS-to-total-asthma-medication ratios from primary care electronic medical records (EMRs) in detecting patients at risk of asthma exacerbation, as approached by prescription of oral corticosteroids and/or antibiotics.

**Methods:**

Retrospective cohort studies were identified, using the Health Improvement Network general practice database (THIN, United Kingdom) and the Cegedim Longitudinal Patient Data (France). We selected asthma patients aged 16–40 years, with ≥ 4 prescriptions for asthma medications in 2007 and ≥ 1 prescription in 2008. For each country, three groups were defined according to ratio value in 2008: 0% (non-ICS users), <50% (low-ICS-ratio group) and ≥50% (high-ICS-ratio group). Outcomes were marker of asthma exacerbations: systemic corticosteroids and antibiotics. They were compared between groups in each country.

**Results:**

Among 38,637 British and 4,587 French patients, higher numbers of prescriptions per patient of systemic corticosteroids, antibiotics and total asthma medications were observed in the low-ICS-ratio groups compared to other groups (p < 0.0001 for each outcome in both countries). Likewise, low-ICS-ratio patients had more medical contacts (p < 0.0001 in both countries), suggesting poorly controlled asthma. ICS-treated patients had lower risks of receiving systemic corticosteroids in 2008 in the high-ICS-ratio group, compared to the low-ICS-ratio group: RR = 0.54, 95%CI = [0.50-0.57] and RR = 0.78, 95%CI = [0.67-0.91] in the UK and France, respectively.

**Conclusions:**

Patients with high ICS-to-total-asthma-medication ratios presented fewer asthma-related outcomes. The low ICS-to-total-asthma-medication ratio calculated with EMRs data reflects insufficient prescribing of ICS relative to all asthma medications, which may lead to deteriorated asthma control.

## Background

The controller-to-total-asthma-medication ratio has been used to assess the quality of asthma care using the United States claims data [1-3]. Patients with controller-to-total-asthma-medication ratio values of 50% and higher exhibited fewer asthma exacerbations than those with lower ratios, as assessed by proxies such as hospitalizations or visits to emergency departments [1,3]. Consistent results were also obtained after restraining controllers to inhaled corticosteroids (ICS) and calculating ICS-to-total-asthma-medication ratios using French claims data [4]. To our knowledge, controller-to-total-asthma-medication ratios and particularly the ICS-to-total-asthma-medication ratio have not been computed using prescribing data from primary care electronic medical records (EMRs), though other ratios have been studied, such as ICS-to-reliever ratios [5,6] and controller-to-reliever ratios [7]. Further, the distributions within the low and high ICS-to-total-asthma-medication ratio subgroups have been little explored. It is unclear whether these distributions are homogenous, or if distinct subgroups with specific characteristics can be identified within high and/or low-ICS-ratio groups.

Prescribing data reflect health care professional’s (HCP) diagnoses and decisions and, therefore, provide information that is complementary to claims data, which result from both HCP’s prescribing and patients’ compliance with prescription orders. Hence, using prescribing data, it is possible to assess if high-ICS-ratio patients present fewer asthma exacerbations than those with lower ratio values. Further, as prescribing may be affected by the local organization of health care, e.g. national prescribing guidelines, it was of interest to explore the relationship between ratios and medical resource utilisation in countries with two different health care systems, i.e. France and the United Kingdom (UK). In the United Kingdom, GPs are employed by the NHS or by general practices whereas most French primary care physicians have a liberal status, and their incomes primarily depend on the volume of their activity. In contrast, the quality of care impacts British physicians’ salary due to the presence of a pay for performance scheme***.*** This system rewards general practices that achieve targets set over a wide range of clinical indicators. For instance, physicians are encouraged to perform annual disease reviews for their asthma patients, which include an assessment of disease control [8].

Our objective was to determine whether ICS-to-total-asthma-medication ratios, computed in primary care prescribing data, could be used to detect patients at risk of asthma exacerbation, by comparing prescriptions and medical contacts between non-ICS users, low- and high-ICS-ratio groups.

## Methods

### Study design and data sources

We conducted a database study using primary care electronic medical records data from the UK and France (The Health Improvement Network or THIN and Longitudinal Patient Data or LPD). The data between 1st January 2007 and 31th December 2008 were extracted for all patients with at least one prescription in 2007 of respiratory drug ATC: R03. Extractions included patients’ age, gender, and information on general practitioner visits and medication prescribing in 2007 and 2008.

THIN collects anonymized longitudinal primary care patient records including demographic, medical and prescription information at an individual patient level from participating general practices in the UK that routinely record these data. The data collection was approved by the NHS South-East Multi-centre Research Ethics Committee in 2003. The THIN data were shown to be generalizable to the UK population [9,10].

To derive data for the French patients, the LPD database was used. The database includes anonymous data on patient consultations and prescribed medications. General practitioners belonging to the LPD Network are representative of the French GP population based on age, gender and geographical region. LPD holds an ethical approval from the French National Data Protection Authority (CNIL) for data collection since 2002. As our study utilized anonymized patients data only, no additional ethical approval was needed.

### Cohort selection

We received extractions of patients with at least one prescribed asthma drug (ATC Classification: R03) in 2007, from THIN (United-Kingdom) and the LPD database (France). From these data, we selected patients aged between 16 and 40 years who received at least 4 prescriptions for asthma medications in 2007 [11] and at least one prescription for an asthma medication in 2008. We excluded patients receiving tiotropium during the study time frame. Exclusion criteria were used to limit the presence of patients with chronic obstructive respiratory disease (COPD). Differentiation of asthma and COPD can be challenging in primary care. Additionally, both diseases are mostly treated by the same drug classes. However, COPD typically occurs after the age of 40 and tiotropium was one of the few respiratory drugs specific to COPD at the time of the study.

### ICS-to-total-asthma-medication ratio (ICS/R03)

Respiratory medications included short-acting beta-2-adrenoreceptor agonists (SABA), anticholinergics, SABA/anticholinergic fixed combinations, long-acting beta-2-adrenoreceptor agonists (LABA), inhaled corticosteroids (ICS), LABA/ICS fixed combinations, leukotriene receptor antagonists (LTRA), xanthines and cromones.

The ICS-to-total-asthma-medication ratio constituted the proportion of prescribed units of ICS out of the overall number of respiratory medication units [11] prescribed during 2008. In French data, we employed units of prescribed medications (i.e., 1 unit = 1 canister for inhaled therapy or 1 packaging for oral therapy) in the calculation of the ratios as patients can receive more than one canister/package with one prescription in France. As the number of prescribed units was not available in the THIN data for oral therapy, the ICS-to-total-asthma-medication ratio in the UK was computed based on the number of prescriptions recorded. In both countries, LABA/ICS fixed combinations and not combined ICS were all counted as one ICS unit in ratios, both in numerator and denominator. Three groups were defined according to the value of ICS-to-total-asthma-medication ratio in 2008: R = 0% (non ICS users), 0% < R < 50% (low-ICS-ratio group) and R ≥ 50% (high-ICS-ratio group).

### Outcomes

The outcomes were prescriptions for systemic corticosteroids and respiratory antibiotics (beta-lactams, first to third generation cephalosporins, macrolides and fluoroquinolones). All outcomes were assessed from 1 January 2008 to 31 December 2008.

### Statistical analyses

We analysed the data from the UK and France according to the same statistical analysis plan, in four successive steps.

- Distribution of ratios and definition of groups
  The distribution of the ICS-to-total-asthma-medication ratios was first examined. The patients were then assigned into the ratio groups (non ICS users, low-ICS-ratio group and high-ICS-ratio group, as defined in the specific paragraph).

b. Univariate analyses
  Then, the different groups of patients were compared according to patients’ age and gender, number of GP visits, and the total number of prescribed asthma medications in 2008 (marker of asthma severity), and the systemic corticosteroids and antibiotics prescribing levels (outcomes). Chi^2^ test and ANOVA were used for univariate analyses.

c. Multivariate analyses
  Multivariate logistic regression models were then conducted among ICS-treated patients only (low and high-ICS ratio groups) to determine the adjusted risk of receiving systemic corticosteroids (respiratory antibiotics) according to ratio value (high vs. low). Adjustments were made for age, gender, use of a fixed LABA-ICS combinations in 2008, and the number of respiratory medications prescribed in 2008 (<6, ≥ 6). For all models, the low-ICS-users group was used as the reference.

d. Sensitivity analyses
  Sensitivity analyses on prescriptions for systemic corticosteroids and antibiotics in patients receiving ≥ 6 prescribed units of respiratory medications (≥6 prescriptions in the UK) were also performed in univariate analyses, as some ratios may be high as a consequence of low prescribing levels of respiratory medications (small denominator). Furthermore, in case of clearly distinguishable subgroups within the low, or within the high-ICS-ratio group in the main analysis, complementary comparisons between the different subgroups were performed in the given group according to patient number of medical contacts, studied outcomes and prescribed asthma medications in 2008.

All analyses were performed using SAS statistical software, version 9.2 (SAS Institute, Cary, North Carolina). All reported p-values are 2-tailed, with significance level set at 5%.

## Results

**Figure 1:**
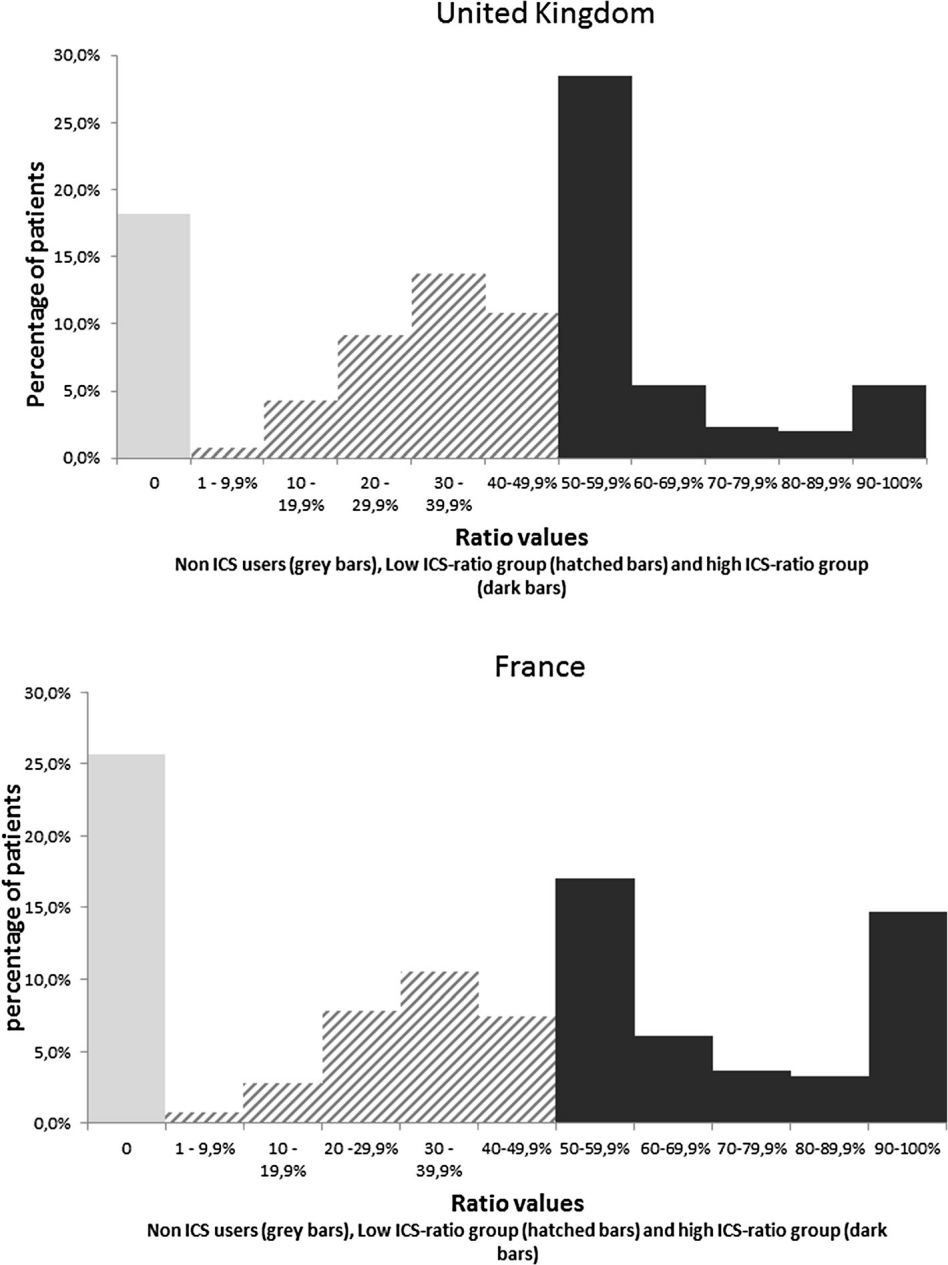
Distribution of ICS-to-total-asthma-medication ratio in the UK (n = 38,637) and in France (n = 4,587).

The extractions of patients under respiratory therapy in 2007 included 343,156 patients in the UK and 119,265 in France. After applying inclusion criteria, we identified in the extractions 38,637 patients (11.2%) in the UK THIN data (mean age = 29 years, 53% females) and 4,587 patients (3.8%) in the French LPD data (mean age = 28 years, 54% females). The ratio distributions for the UK and France are presented in Figure 1.

The shape of the observed ICS-to-total-medication ratios distribution was similar in both countries; however there were differences in the proportion of non ICS users (26% in France vs. 18% in the UK). In the low-ICS-ratio groups, the distributions presented a Gaussian-like pattern in both countries, with a maximal frequency in the 30–39.9% interval.

More than 40% of British and French patients belonged to the high-ICS-ratio group (43.3% and 44.7%, respectively) and three distinct sub-groups were identified within this group in both countries. We observed a clear distribution peak right after the 50% threshold, in the 50–59.9% interval. These patients accounted for 65% of the high-ICS-ratio group in the UK and 38% in France. Another sub-population could be identified for the highest ratio values, the 90% and greater, notably in France (33% of the high-ICS-ratio group), and to a lesser extent, in the UK (12% of this group).

The medical resource utilization data for the three groups are presented in Table 1 and Table 2. The groups were found to be similar in age and gender. The patients in the low-ICS-ratio group received more prescriptions for systemic corticosteroids and antibiotics and visited GPs more frequently. When the analyses were restricted to patients with ≥6 prescriptions in the UK (and ≥ 6 prescribed units in France) in 2008, the findings were consistent with the main analyses. Systemic corticosteroids and LABA/ICS fixed combinations were found to be prescribed more frequently in France than in the UK.

We were able to identify three subgroups in the high-ICS-ratio groups (50–59.9%, 60%-89.9%, and ≥90%). These subgroups were compared in terms of number of GP visits and prescribed therapy. In both countries, patients with the highest ratio values (≥90%) were prescribed fewer systemic corticosteroids and antibiotics as well as asthma medications, more markedly in the UK, where these patients also visited their GPs less frequently. The differences between the 50–59.9% and 60–89.9% subgroups, however, were less pronounced for systemic corticosteroids. We did not observe any apparent uniform downward trend in number of systemic corticosteroids and antibiotic prescriptions in the three subgroups as the ratio increased (Table 3).

In the multivariate main analyses performed on the UK data, the high-ICS-ratio group had a significantly lower risk of receiving a prescription for systemic corticosteroids or antibiotics than the low-ICS-ratio group. The conclusions for French patients were similar, suggesting that there was an association between the high-ICS-to-total-asthma-medication ratio and lower number of systemic corticosteroids prescriptions, while decreased risks of receiving antibiotics were also observed. It approached significance in France (Table 4).

**Table 1:** **Medical resource utilization and prescribed medications in 2008 (United Kingdom)**

	**R = 0%**	**0% < R < 50%**	**R ≥ 50%**	**p-value**
***All patients***				
**Number of patients**	6,996	14,903	16,738	
**Age, years (mean)**	28.9	29.4	29.4	<0.0001
**Gender, female (%)**	49.6%	53.8%	55.1%	<0.0001
**Number of GP visits (mean)**	5.5	7.7	6.6	<0.0001
**Prescribed therapy**				
**Number of respiratory medication prescriptions (mean)**	3.6	12.9	6.9	<0.0001
**Patients with ≥ 1 prescription of LABA-**	-	41.1%	44.3%	<0.0001
**ICS fixed combination (%)**				
**Number of short-acting beta agonists prescriptions (mean)**	3.4	6.7	2.7	<0.0001
**Systemic corticosteroids**				
**Patients with ≥ 1 prescription (%)**	6.2%	22.5%	12.7%	<0.0001
**Number of prescriptions (mean)**	0.1	0.5	0.2	<0.0001
**Antibiotics** ^**(a)**^				
**Patients with ≥ 1 prescription (%)**	34.5%	47.9%	41.4%	<0.0001
**Number of prescriptions (mean)**	0.7	1	0.8	<0.0001
***Patients with at least 6 prescriptions of respiratory medications in 2008***				
**Number of patients**	1,384	11,835	8,391	
**Systemic corticosteroids**				
**Patients with ≥ 1 prescription (%)**	8.2%	24.6%	15.5%	<0.0001
**Number of prescriptions (mean)**	0.2	0.5	0.3	<0.0001
**Antibiotics** ^**(a)**^				
**Patients with ≥ 1 prescription (%)**	35%	49.3%	44.8%	<0.0001
**Number of prescriptions (mean)**	0.8	1.1	0.9	<0.0001

^(a)^Beta-lactams, cephalosporins (first to third generation), macrolides and fluoroquinolones.

**Table 2:** **Medical resource utilization and prescribed medications in 2008 (France)**

	**R = 0%**	**0% < R < 50%**	**R ≥ 50%**	**p-value**
***All patients***				
**Number of patients**	1,176	1,358	2,053	
**Age, years (mean)**	28.1	28.4	29.5	<0.0001
**Gender, female (%)**	54.7%	53.5%	55%	0.66
**Number of GP visits (mean)**	5.3	6.5	5.6	<0.0001
**Prescribed therapy** ^**(a)**^				
**Number of respiratory medication units (mean)**	5.1	18.6	8.7	<0.0001
**Patients with ≥ 1 prescription of LABA-ICS fixed combination (%)**	-	71.2%	81.2%	<0.0001
**Number of short-acting beta agonists units (mean)**	2.9	6	1.8	<0.0001
**Systemic corticosteroids**				
**Patients with ≥ 1 prescription (%)**	21.9%	36.1%	30.5%	<0.0001
**Number of units (mean)**	0.6	1.2	0.8	<0.0001
**Antibiotics** ^**(b)**^				
**Patients with ≥ 1 prescription (%)**	46.5%	55.7%	53%	<0.0001
**Number of units (mean)**	1.3	2.1	1.7	<0.0001
***Patients with at least 6 prescriptions of respiratory medications in 2008***				
**Number of patients**	367	1,153	1,152	
**Systemic corticosteroids**				
**Patients with ≥ 1 prescription (%)**	21%	36%	32.1%	<0.0001
**Number of units (mean)**	0.7	1.2	1	0.05
**Antibiotics** ^**(b)**^				
**Patients with ≥ 1 prescription (%)**	43.9%	55.3%	52%	0.0006
**Number of units (mean)**	1.3	2	1.8	<0.0001

^(a)^Canisters for inhaled therapy and packages for oral therapy in France.

^(b)^Beta-lactams, cephalosporins (first to third generation), macrolides and fluoroquinolones.

**Table 3:** **Complementary analyses: Medical resource utilization and prescribed medications in 2008 in the 3 subgroups identified within the high-ICS-ratio group**

	**50% ≤ R < 60%**	**60% ≤ R < 90%**	**90% ≤ R**	**p-value**
**UK**				
**Number of patients**	10,929	3,715	2,094	
**Number of respiratory medication prescriptions (mean)**	7.5	6.86	3.62	<0.0001
**Systemic corticosteroids**				
**Patients with ≥ 1 prescription (%)**	12.7%	14.7%	8.9%	<0.0001
**Number of prescriptions (mean)**	0.2	0.24	0.16	0.0006
**Antibiotics** ^**(a)**^				
**Patients with ≥ 1 prescription (%)**	41.9%	43.4%	35.5%	<0.0001
**Number of prescriptions (mean)**	0.8	0.86	0.7	0.0001
**Patients with ≥ 1 prescription of LABA-ICS fixed combination (%)**	38.9%	56.2%	51%	<0.0001
**Number of GP visits (mean)**	6.5	7.2	5.9	<0.0001
**France** ^**(b)**^				
**Number of patients**	783	597	673	
**Number of respiratory medication prescriptions units (mean)**	9.61	11	5.67	<0.0001
**Systemic corticosteroids**				
**Patients with ≥ 1 prescription (%)**	29.9%	33.3%	28.7%	0.178
**Number of units** ^**(b)**^ **(mean)**	0.84	0.82	0.77	0.811
**Antibiotics** ^**(a)**^				
**Patients with ≥ 1 prescription (%)**	50.7%	55.8%	53.3%	0.17
**Number of units** ^**(b)**^ **(mean)**	1.61	1.91	1.52	0.0159
**Patients with ≥ 1 prescription of LABA-ICS fixed combination (%)**	75.5%	89.9%	82%	<0.0001
**Number of GP visits (mean)**	5.4	5.8	5.6	0.273

^(a)^Beta-lactams, cephalosporins (first to third generations), macrolides and fluoroquinolones**.**

^(b)^Canisters for inhaled therapy and packages for oral therapy.

## Discussion

In this database study utilizing primary care data we calculated ICS-to-total-asthma-medication ratios and assessed asthma related outcomes in more than 40,000 patients from the UK and France. Although not identical, the distributions of therapeutic ratios presented similarities in both countries, with a Gaussian-like pattern in the low-ICS-ratio groups. Patients untreated by ICs were more numerous in France. In both countries, patients in the low-ICS-ratio group (<50%) were prescribed more systemic corticosteroids, antibiotics and asthma medications than patients from other groups. These patients also visited their GPs more frequently than the high-ICS-ratio group.

Our findings are consistent with prior research on the controller-to-total-asthma-medication ratios performed in claims data using asthma-related hospital contact as outcome [1,3]. A lower risk of being dispensed systemic corticosteroids was also observed in patients with high-ICS-to-total-medication ratios compared to those with lower ratios [4]. Also, these findings are in line with a better asthma control, as observed in patients with higher ratios (≥50%) by validation studies [12,13].

The interpretation of ratio values computed from primary care prescribing data differs from that of claims data. A high ratio in prescribing data may not reflect patients’ actual exposure to ICS, as prescriptions not dispensed by community-pharmacies are included in analyses. Conversely, unlike claims databases, a low ICS-to-total-asthma-medication ratio in primary care data is fully attributable to insufficient prescribing of ICS by physicians, irrespective of patients’ behaviour, except in case of fragmented care.

Our findings suggest that some GPs insufficiently prescribe ICS and that such prescribing patterns may facilitate asthma exacerbations [14]. Utilising General Practice records to identify patients that receive insufficient ICS treatment relative to overall asthma therapy and introducing corrective interventions can, therefore, help decrease the burden of asthma.

The guidelines [15,16] recommend a daily use of controllers in persistent asthma, particularly ICS. Patients untreated by ICS were more numerous in France, which might be explained by a poorer adherence to the guidelines than in the UK. However, it could also be that some non-ICS patients might have intermittent or milder asthma while others might be treated with LTRAs monotherapy. The non-ICS group was characterised by a lower number of prescriptions for systemic corticosteroids as well as for asthma medications.

In patients receiving ICS, we selected the 50% ratio value as a cut-off to classify the patients into the low-ICS-ratio and the high-ICS-ratio group. Altogether, more than one third of all patients were in the low-ICS-to-total-asthma-medication group. Patients from this group were prescribed more asthma medications, systemic corticosteroids, and they visited their GP more frequently. Our multivariate models suggested that, compared to these patients, the high-ICS-ratio group presented lower risks of both receiving systemic corticosteroids and antibiotics (Table 4). It cannot be excluded that the increased resource utilisation in the low-ICS ratio may be partly due to a higher severity of asthma, as suggested by the higher number of asthma medications prescribed to this group. Also, our findings were not substantially affected after restricting the analyses to patients with at least 6 prescribed units of respiratory medications (prescriptions in the UK), confirming the robustness of our findings. Additionally, as ratio denominator comprises all respiratory medications prescribed during the 12-month period, the severity of asthma, as measured by intensity of treatment, is partially accounted for [2,17], thus further limiting the risk of an indication bias.

**Table 4:** **Logistic regression models: Risks of receiving in 2008 ≥ one prescription of systemic corticosteroids (model 1) and risk of receiving ≥ one prescription of antibiotics (model 2), according to ratio groups in ICS-treated patients (non ICS users excluded)**

	**Model 1: Risks of receiving in 2008 ≥ one prescription of systemic corticosteroids**		**Model 2: Risks of receiving in 2008 ≥ one prescription of antibiotics**	
	**Adjusted OR** ^**(a)**^	**95% CI**	**Adjusted OR** ^**(a)**^	**95% CI**
**UK (n = 31,641)**				
**Low ratio (0% < R < 50%)**	1	-	1	-
**High ratio (R ≥ 50%)**	0.54	0.50-0.57	0.80	0.76-0.84
**France (n = 3,411)**				
**Low ratio (0% < R < 50%)**	1	-	1	-
**High ratio (R ≥ 50%)**	0.78	0.67-0.91	0.87	0.75-1.01

^(a)^Adjusted for age, gender, ≥ 1 dispensing of LABA-ICS fixed combination, the number of dispensed respiratory medication classes in 2008 (<6, 6+).

The low-ICS-ratio patients also tended to be prescribed fewer fixed LABA-ICS fixed combinations, particularly in France. Patients prescribed fixed LABA-ICS combinations may require less SABA and non-combined LABA which in turn decreases the ratio denominator. This higher use of fixed combinations in the high ICS ratio group has been observed previously [18].

Upon examining the distribution within the high-ICS-ratio group we identified three subgroups (50–59.9%, 60–89.9%, ≥90%). In France the three subgroups did not differ significantly from one another in terms of systemic corticosteroids, antibiotics prescribing and medical visits. In the UK, however, the ≥90% subgroup patients received fewer systemic corticosteroids and antibiotics, while they visited their GPs less frequently. Also, the number of prescriptions for all respiratory medications was lower in patients in this subgroup with the highest ratios (≥90%), both in the UK and in France, which could suggest a milder asthma or less regular prescribing. The 90% threshold, however, is not clinically meaningful in assessing the quality of care as only a marginal proportion of patients with a milder disease can be identified. Therefore, our data supports the choice of the 50% threshold value, as the outcomes in the three high-ICS-ratio subgroups showed no steady trend towards improvement in asthma control with increasing ICS-to-total-asthma-medication ratio. A possible direction for further research will be to bring together primary care prescribing data, medication dispensation data and patient-reported data to determine the best cut-off values for ICS-to-total-asthma-medication ratio threshold.

We were not able to use an identical ratio definition in both countries due to differences in the data on prescribed medications. The UK data did not contain information allowing counting the number of prescribed packages for oral therapy. We therefore calculated the ratios for the UK patients using the number of ICS prescriptions in the numerator and the number of all respiratory medication prescriptions in the denominator. However, this potential bias should not have affected the validity of this definition as both the numerator and denominator were similarly approximated. Despite these discordant definitions, consistent results were obtained between the two countries. Our definitions of ratio did not take into account the dosing of ICS canisters nor the potency of the molecule, hence can be considered ‘unweighted’. In comparison with a more elaborate alternative [19], this turned out to be more discriminating, as severe patients at a higher risk of asthma exacerbations tend to receive more potent ICS and, therefore, would likely qualify in the high-ICS ratio group. Further, the ratio that can be obtained by a more basic calculation has a practical benefit as it can be computed in practice without elaborate programming.

Our study has some limitations. As we wanted to include patients receiving regular prescribing for asthma, our study population corresponded to a minority of selected persistent asthmatics with a close supervision in primary care. Thus, these patients were not representative of the overall population of asthmatics. Prescriptions from physicians not affiliated to the study networks were not available in the database. Henceforth, the number of prescriptions might have been underestimated if fragmented care or partial supervision by specialists did occur. Nevertheless, we believe that this bias may not affect our conclusions. Firstly, analyses were conducted in patients with at least four annual prescriptions of asthma medications during baseline period (2007). Then, the underestimation of prescriptions should be similar in both numerator and denominator of therapeutic ratios. As another limitation, major outcomes such as hospital admissions or emergency room visits were not available in these databases. We used proxies, such as systemic corticosteroids and antibiotics prescriptions, to identify asthma exacerbations. Unlike systemic corticosteroids, antibiotics however are not listed as outcomes for asthma [20,21], though they are commonly used in asthma [22,23]. Nonetheless, the consistency of our data on systemic corticosteroids and antibiotics support the validity of our results. Finally, the ICS-to-total-asthma-medication ratio did not take into account leukotriene-receptor antagonists. Our preceding studies nonetheless showed that the addition of LTRA to ICS in the numerator yielded close conclusions regarding the ability to detect at-risk patients [4,13].

Based on these findings, it should be of interest to verify whether patients of the low ratio group are more likely to be supervised by specific profiles of GPs. In such case, the reasons that lead these physicians to prescribe a limited amount of ICS compared to other anti-asthma drugs needs to be better understood. Then, targeted educational campaigns should be carried out in primary care to improve the quality of prescribing in asthma, specifically among low prescribers of ICS.

## Conclusions

In conclusion, our findings suggest that insufficient prescribing of ICS in primary care may lead to asthma exacerbations and, consequently, increased medical resource utilization. Quality of care could improve if the ICS-to-total-asthma-medications ratios were used to screen irregular use of ICS in asthma before potential occurrence of adverse outcomes.

## Abbreviations

ATC: Anatomical Therapeutic Chemical (classification)
LPD: Longitudinal Patient Data
EMRs: Electronic medical records
HCP: Health care professional
ICS: Inhaled corticosteroids
LABA: Long-acting beta-2-adrenoreceptor agonists
LTRA: Leukotriene receptor antagonists
SABA: Short-acting beta-2-adrenoreceptor agonists
UK: United Kingdom
