# Erratum to: Prescribed therapy for asthma: therapeutic ratios and outcomes

**DOI:** 10.1186/s12875-016-0404-4

**Published:** 2016-01-25

**Authors:** Laurent Laforest, Idlir Licaj, Gilles Devouassoux, Irene Eriksson, Pascal Caillet, Gérard Chatte, Manon Belhassen, Eric Van Ganse

**Affiliations:** Pharmacoepidemiology Lyon, UMR 5558 CNRS - Claude Bernard University, Lyon, France; Respiratory Medicine, Croix Rousse University Hospital, Lyon, France; Centre for Pharmacoepidemiology, Department of Medicine, Karolinska Institutet, Stockholm, Sweden; Epidemiology and Public Health Department, Amiens University Hospital Center, Amiens, France; Respiratory physician, Caluire, France

Unfortunately, the original version of this article [1] contained an error. The name of one of the authors was included incorrectly and it read “Eric Ganse” instead of “Eric Van Ganse”.

The name has been updated in the original article and is also correctly included in full in this erratum.
